# Unfallchirurgisch Relevantes zu Bissverletzungen von Mensch und Tier

**DOI:** 10.1007/s00113-024-01441-1

**Published:** 2024-06-17

**Authors:** Katharina Estel, Catharina Scheuermann-Poley, Ole Goertz, Jennifer Urban, Kristina Landscheidt, Werner Wenzel, Christian Willy

**Affiliations:** 1 Klinik für Unfallchirurgie, Orthopädie und Septisch-Rekonstruktive Chirurgie, Bundeswehrkrankenhaus Berlin, 10115 Berlin, Deutschland; 2 Klinik für Plastische, Rekonstruktive & Ästhetische Chirurgie, Handchirurgie, Martin Luther Krankenhaus und Evangelische Elisabeth Klinik Berlin, Berlin, Deutschland; 3 Klinik für Mikrobiologie, Bundeswehrkrankenhaus Berlin, Berlin, Deutschland

**Keywords:** Wunden, Wundinfektion, Débridement, Antibiotikatherapie, Postexpositionsprophylaxe, Wounds, Debridement, Antibiotic therapy, Wound infection, Postexposure prophylaxis

## Abstract

Bissverletzungen stellen wegen des tief inokulierten polymikrobiellen Erregerspektrums, möglicher Begleitverletzungen und ausgeprägter Weichteilschäden ein differenziert zu behandelndes Krankheitsbild dar. Hundebisse sind die häufigsten Bissverletzungen und heilen, verglichen mit Menschen- und Katzenbissen, weniger komplikativ. Die Bisslokalisation hängt stark vom Alter und von der Größe des Bissopfers sowie der Art des Bissverursachers ab. Bei jedem Biss ist der Erregernachweis anzustreben, um auf eine Exazerbation kalkuliert reagieren zu können. Die primäre Antibiotikatherapie sollte empirisch mit Amoxicillin + Clavulansäure oder Ampicillin + Sulbactam erfolgen und ggf. im Verlauf antibiogrammgemäß angepasst werden. Die chirurgische Sanierung beinhaltet je nach Befund das Ausschneiden des Bisskanals und das differenzierte Wund-Débridement. Wichtig sind die Überprüfung des Impfstatus der Beteiligten und, wenn indiziert, die Postexpositionsprophylaxe für Tetanus und Tollwut.

## Abstract (en)

Bite injuries require differentiated treatment due to the deeply inoculated polymicrobial pathogen spectrum, possible concomitant injuries and pronounced soft tissue damage. Dog bites are the most common bite injuries but are less complicated to heal than human and cat bites. The location of the bite greatly depends on the age and the size of the bite victim as well as the type of bite perpetrator. In every case detection of the pathogen should be carried out to provide the best possible adapted treatment in the event of an exacerbation. The primary antibiotic treatment should be empirical with amoxicillin + clavulanic acid or ampicillin + sulbactam and, if necessary, adjusted according to the antibiogram. Depending on the findings, surgical treatment includes excision of the bite canal and a customized wound debridement. It is important to check the vaccination status of those involved and if indicated, to carry out postexposure prophylaxis for tetanus and rabies.

## Lernziele

Nach der Lektüre dieses Beitrags

- können Sie die Relevanz der Tier- und Menschenbisse einschätzen.
- ist es Ihnen möglich, die primären Versorgungsstrategien festzulegen.
- sind Sie in der Lage, die optimale antibiotische Therapie einzuschlagen.
- kennen Sie Grundzüge der chirurgischen Versorgung.
- erkennen Sie Komplikationen der Bissverletzungen zuverlässig.

## Einleitung

Bissverletzungen verursachen in den USA ca. 1 % der Notaufnahmevorstellungen [1]. Trotz der vergleichsweise geringen Anzahl schwerer Verletzungen stellen sie für den Chirurgen durch die tiefe Kontamination mit ungewohnten Erregern, bei kleinen Wunden durch die schwierige Beurteilung von Wundtiefe und -ausmaß sowie bei großen Wunden durch die Notwendigkeit einer schon frühzeitig oft auch interdisziplinär zu planenden plastisch-rekonstruktiven Versorgung eine Herausforderung dar [2]. Vor diesem Hintergrund wird im Folgenden auf die unterschiedliche Gefährlichkeit, übliche Prädilektionsstellen der Bissverletzungen, alters- und geschlechtsspezifische Eigenarten, typische Komplikationen, die erforderlichen diagnostischen und therapeutischen Maßnahmen sowie die zu berücksichtigenden mikrobiologischen Aspekte wie Gewebeprobenentnahme, Antibiotikatherapie und ggf. übertragene Infektionskrankheiten im Detail eingegangen.

## Epidemiologie und Verletzungsmuster

### Tierbissverletzungen

Für Deutschland gibt es keine konkreten Zahlen von Bissverletzungen, da keine Meldepflicht vorliegt [3]. Jedoch ist bekannt, dass die Mehrzahl der Tierbisse von Hunden und Katzen ausgeht [3]. Für die USA ließen sich Inzidenzen bis zu 200 Tierbisse auf 100.000 Einwohner pro Jahr nachweisen [4] mit steigender Zahl durch die Zunahme häuslich gehaltener Tiere [5].

Hundebisse stellen mit 60–90 % die häufigste Form der Bissverletzungen dar [1, 6]. Gefolgt werden diese von Katzenbissen mit 5–10 % und seltener von Pferden‑, Schlangen- und Nagetierbissen [1, 3]. Menschen mit viel Tierkontakt, wie **Tierbesitzer**

Tierbesitzer

oder auch **Tierärzte/-pfleger**

**Abb. 1:**
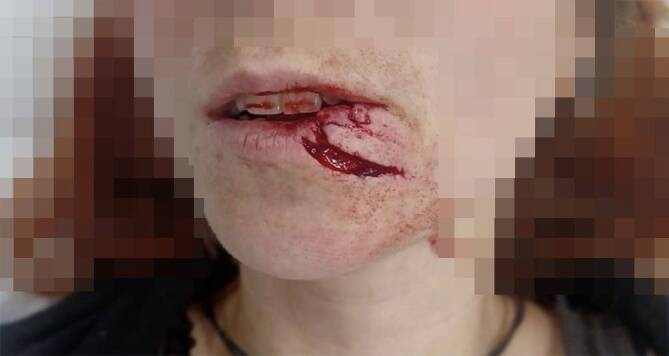
Leicht klaffende Risswunde durch einen Hundebiss, nachdem das Tier durch seine Pflegerin geweckt worden war

Tierärzte/-pfleger,

sind am häufigsten betroffen (Abb. 1). Das männliche Geschlecht wird bevorzugt von Hunden, das weibliche Geschlecht eher von Katzen gebissen [4]. Tierbisse entstehen häufig durch eine Störung des Kontakts zu dem Tier – das Tier wird bedrängt, beim Fressen behindert oder erschreckt [3]. Infolge der instinktiven Abwehrbewegung ist oft die dominante Hand des Opfers betroffen [2]. Todesfälle sind selten. In Deutschland wurden 2019 nur 3,3 Personen durch Hundebisse tödlich verletzt [7].

#### Merke

Störungen des Kontaktes zum Tier können Auslöser für das Beißen des Tieres sein.

#### Hundebissverletzungen

Hundebisse führen bevorzugt zu **Rissverletzungen**

Rissverletzungen

oder **Abrissverletzungen**

Abrissverletzungen

mit Möglichkeit der knöchernen Beteiligung in Abhängigkeit von der Größe und Kraft des Tieres. Große Bisswunden sind in der Mehrzahl der Fälle unregelmäßig geformt, unterminiert, zeigen Kulissen und gehen mit einer **Gewebequetschung**

Gewebequetschung

einher [8]. Je nach der Hunderasse können die Verletzungen schwerwiegend sein und eine operative Rekonstruktion erfordern. Hundebisse sind mit **Narbenbildung**

Narbenbildung

verbunden und bergen die Gefahr von lokalen und systemischen **Infektionen**

Infektionen

**Abb. 2:**
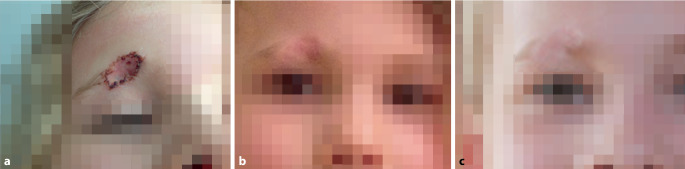
**a** Versorgte Hundebissverletzung eines 5‑jährigen Mädchens durch einen Mischlingsrüden im Gesicht mithilfe ipsilateraler retroaurikulärer Vollhautentnahme nach initialer Wundversorgung. Zwei Wochen alter Befund. **b,** **c** Verlaufsbilder nach einem und 6 Jahren. Die Versorgung der Augenbrauenverletzung mit einer Spalthaut erscheint kosmetisch nicht ideal. Zu berücksichtigende Alternativen sind die Verwendung behaarter Kopfhaut und die Serienexzision im Verlauf

[5, 8]. Die Lokalisation der Hundebissverletzung hängt vom Alter bzw. von der Größe der verletzten Person ab. Erwachsene haben bevorzugt Verletzungen der Extremitäten, Kinder Verletzungen des Kopfes ([6, 9, 10]; Abb. 2). Für die **offenen Gesichtsverletzungen**

offenen Gesichtsverletzungen

kann die Stadieneinteilung nach Lackmann herangezogen werden ([11]; Tab. 1). In bis zu 75 % aller Verletzungen sind männliche Hunde involviert. Begünstigende Faktoren sind eine genetische Veranlagung zu Aggressivität, Gesundheitszustand sowie mangelnde Erziehung und Sozialisierung des Hundes [6].

**Tab. 1:** Stadieneinteilung von offenen Hundebissverletzungen im Gesichtsbereich. (Nach Lackmann et al. [11])

Stadium	Klinisches Bild
I	Oberflächliche Verletzung ohne Beteiligung der Muskulatur
II	Tiefe Verletzung mit Beteiligung der Muskulatur
III	Tiefe Verletzung mit Beteiligung der Muskulatur und Substanzdefekt
IVA	Stadium III sowie Gefäß- und Nervenverletzung
IVB	Stadium III und Knochenbeteiligung

##### Merke

Hundebisse sind meist Riss- oder Abrissverletzungen, die je nach Größe der betroffenen Person hauptsächlich die Extremitäten oder die Kopfregion betreffen.

#### Katzenbissverletzungen

**Abb. 3:**
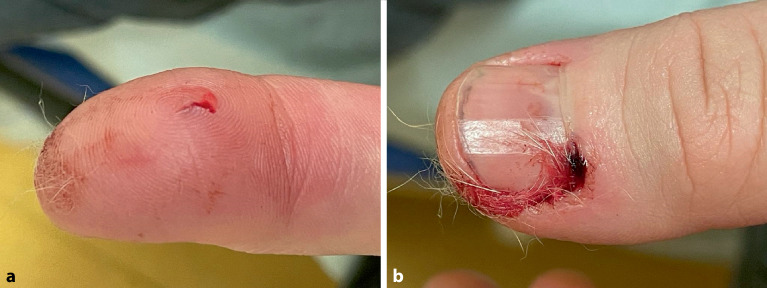
Katzenbissverletzung: punktförmig durch die eigene Katze mit Tierhaarresten in der Wunde. **a** Palmare Ansicht, **b** dorsale Ansicht

Katzen verursachen nach den Hunden die zweithäufigsten Bissverletzungen, die infolge der spitzen Eckzähne der Katzen eher mit punktförmigen kleinen Verletzungen einhergehen ([2]; Abb. 3 und 4). Gehäuft sind die Hände, insbesondere der Zeigefinger betroffen [12, 13]. Primär entwickelt sich innerhalb von wenigen Stunden nach dem Biss eine **Lymphangiosis**

Lymphangiosis

[13] mit den klinischen Symptomen wie Rötung, Ödem und Schmerz [14]. Viele Verletzungen können mit einfachen Maßnahmen komplikationslos ausheilen [12]. Daneben können Bissverletzungen aber auch eine Phlegmone, Tendosynovitis, Arthritis, einen Abszess und eine Septikämie zur Folge haben [13, 14, 15]. Bei Katzen ist die Infektionsrate der Wunden höher (ca. 50 %) als bei Hunden (ca. 25 %) [12]. Katzen haben vergleichsweise spitzere und längere Zähne, sodass die Bakterien des Speichels tiefer in das Gewebe eindringen [2]. An den typischen Prädilektionslokalisationen, Hand und Unterarmen, können sich die **anaeroben Erreger**

anaeroben Erreger

in dem dort häufig betroffenen Sehnen‑, Sehnenscheiden‑, Gelenk- und Gelenkkapselgewebe aufgrund der limitierten nutritiven Versorgung dieser Gewebetypen besonders gut vermehren [3, 16, 17, 18].

**Abb. 4:**
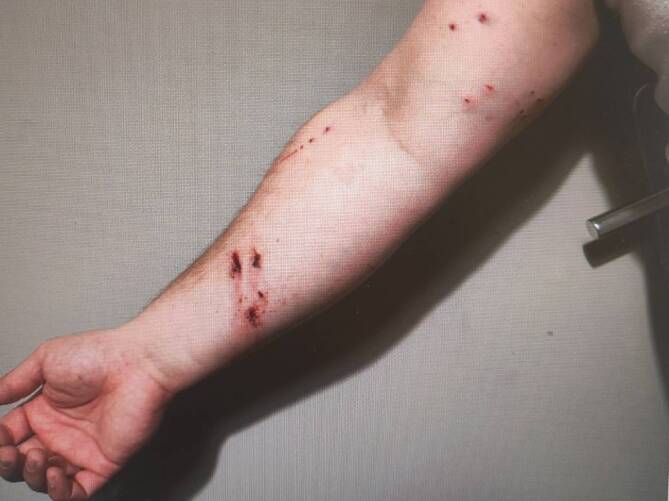
Katzenbissverletzung: punktförmige Bissverletzungen sowie Kratzspuren durch die eigene Katze mit Notwendigkeit der chirurgischen Sanierung sowie i.v.-Antibiotikatherapie

##### Merke

Katzenbisse sind meist punktförmig und haben eine höhere Infektionsrate als Bisse von anderen Tieren.

### Menschenbissverletzungen

Menschbissverletzungen sind insgesamt eher selten und machen nur 1 % der Bissverletzungen aus. In Städten kann ihr Anteil jedoch auf bis zu 20 % ansteigen [4]. Das männliche Geschlecht ist insgesamt häufiger betroffen [19], wobei dies v. a. für die Form der „Fight-bite-clenched-fist-Verletzung“ gilt. Hierbei kommt es zur **„indirekten Bissverletzung“**

„indirekten Bissverletzung“

durch den Schlag der Faust gegen die Zähne. Betroffen sind insbesondere die **Metakarpophalangealgelenke**

Metakarpophalangealgelenke

[19], da diese in der Faustposition am stärksten hervortreten [16]. Die darüber liegende Haut ist zum Zeitpunkt des Aufpralls angespannt, sodass die Weichteile kaum geschützt sind. Die Zähne dringen häufig in den Streckmechanismus und die Gelenkkapsel ein (67 %), was mit einer Verletzung des weniger gut durchbluteten Gelenkknorpels und des Knochens einhergeht [16]. Wenn die Hand anschließend in eine entspannte Position zurückkehrt, wird die verletzte Sehne durch die Retraktion der Strecksehne in der Hautwunde nicht mehr sichtbar und der Kapselriss infolge der **Kulissenverschiebung**

**Abb. 5:**
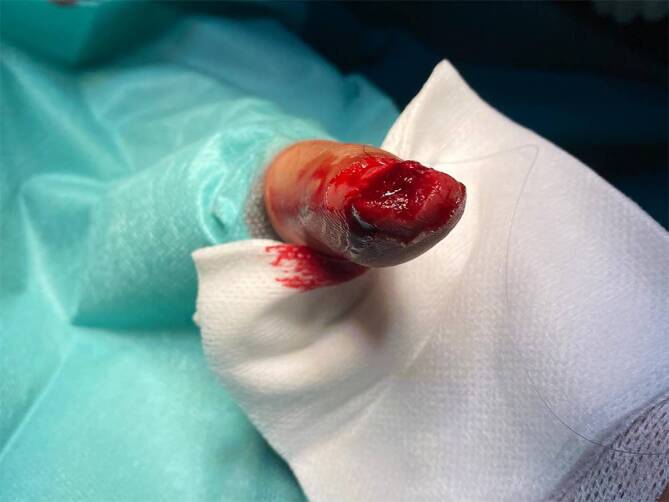
Menschenbissverletzung der Kuppe des Digitus manus 2 im Sinne einer Okklusionsverletzung mit Abriss des Nagels

Kulissenverschiebung

versiegelt. Hierdurch entsteht eine **geschlossene Wunde**

geschlossene Wunde,

die ideal für das Wachstum anaerober Erreger sowie die Entwicklung und großflächige Ausbreitung einer Infektion ist [16]. Infektionsbegünstigend ist außerdem die Tatsache, dass sich diese Patienten meist erst verzögert in der Notaufnahme vorstellen [1, 19]. Demgegenüber stehen Okklusionsbisse, welche infolge einer direkten Verletzung des Weichteilgewebes durch Quetschung zwischen den Zähnen entsteht (Abb. 5). Diese Bisse hinterlassen häufig einen **halbmondförmigen Abdruck**

halbmondförmigen Abdruck

[3]. Ausgeführt werden Okklusionsbisse häufiger durch Männer, betroffen sind vermehrt Frauen insbesondere an Brust, Armen, Genitalien und Beinen [16]. Menschenbisse entstehen im Rahmen von tätlichen Auseinandersetzungen, sexuellen Übergriffen oder bei der Selbstverteidigung, wobei eine hohe Dunkelziffer angenommen wird [16].

#### Merke

Arten der Menschenbisse sind der Okklusionsbiss und die „Fight-bite-clenched-fist-Verletzung“.

## Diagnostik

### Anamnese

Für die Diagnostik sind im Rahmen der Anamnese folgende Parameter wichtig [3]:

- Spezies des Bissverursachers mit Impfstatus der Beteiligten,
- Lokalisation der Bissstelle,
- Art oder der Mechanismus der Verletzung,
- Zeitpunkt der Verletzung, bisherige Primärmaßnahmen und weitere Entwicklung der Wunde.

### Untersuchung und Wundbeschreibung

Im Rahmen der Untersuchung sollten folgende Merkmale beschrieben werden [19]:

- Ausmaß des Schadens,
- Tiefe der Wunde,
- Beteiligung von Strukturen z. B. Sehnen, Gelenke etc.,
- Vorhandensein von Fremdkörpern (Zähne),
- Beweglichkeit betroffener Gelenke.

Es kann sinnvoll sein, die Wunde vorab zu spülen, um bessere Sichtverhältnisse zu erhalten [19]. Dabei dürfen die Erreger nicht mit Druck tiefer in das Weichgewebe gespült werden. **Laboruntersuchungen**

Laboruntersuchungen

mit Bestimmung der **Entzündungsparameter**

Entzündungsparameter

helfen, eine mögliche systemische Beteiligung einzuschätzen [16]. Eine **Sonographie**

Sonographie

zum Ausschluss infektionsbedingter Flüssigkeitsansammlungen und eine konventionelle **Röntgenaufnahme**

Röntgenaufnahme

zum Nachweis knöcherner Läsionen und eingebrachter Fremdkörper, wie z. B. abgebrochener Zähne [16, 20], sind ggf. sinnvoll. Für eine **detaillierte Dokumentation**

detaillierte Dokumentation

empfiehlt sich, die Bissstelle zu fotografieren, bei Menschenbissen zudem den Abstand der Eckzähne des Abdrucks zu bestimmen und ggf. mithilfe eines DNA-Tupfers Speichel des Verursachers zu gewinnen [3]. Diese Maßnahmen können im Rahmen von nachfolgenden rechtlichen Schritten essenziell sein [10].

#### Merke

**Abb. 6:**
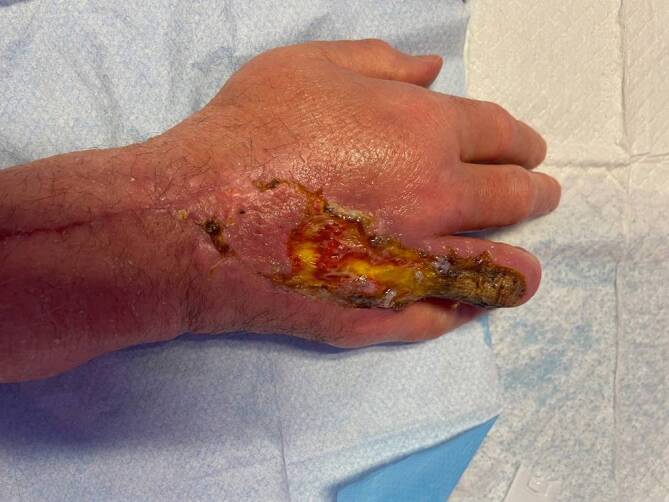
Kobraschlangenbissverletzung nach 3 Monaten: Lokalbefund an rechtsseitigem Handrücken und Ringfinger mit gewebetoxinbedingter chronischer Wundheilungsstörung mit frei liegendem Knochen. Notwendigkeit zur operativen Versorgung mithilfe der Ringfingerexartikulation, des Wund-Débridements über dem 4. Mittelhandknochen und einer Lappenplastik

Ein erhöhtes Infektionsrisiko besteht bei folgenden Wunden:

- tiefe Wunden,
- schwere Gewebezerstörung,
- Wunden an Händen, Füßen, Gesicht und an den Genitalien (Abb. 6),
- Bradytrophik (langsamer Stoffwechsel und langsame Nährstoffversorgung) des verletzten Gewebes (Knorpel, Sehnen), Knochenbeteiligung, Nähe zu Implantaten
- Katzenbissen, gefolgt von Menschenbissen, seltener bei Hundebissen

### Mikrobiologische Diagnostik

#### Erregerspektrum

Das Spektrum der Erreger von Infektionen nach Bissverletzungen rekrutiert sich v. a. aus der residenten und transienten **Mundflora**

Mundflora

des Beißenden, vereinzelt aber auch aus der **Hautflora**

Hautflora

des Gebissenen und Umweltkeimen [21]. Es ist bei Bisswunden grundsätzlich davon auszugehen, dass eine polymikrobielle Kontamination des Wundgebietes vorliegt [22, 23]. Die Breite des Erregerspektrums korreliert mit der Schwere der resultierenden Infektion:

- Bei Hundebissen sind die häufigsten Erreger (30–60 %) *Pasteurella*-Spezies, hämolysierende Streptokokken, *Staphylococcus aureus* sowie Mischinfektionen mit Anaerobiern [23, 24].
- Infektionserreger nach Katzenbissen sind in über 50 % der Fälle *Pasteurella multocida* meist mit aerob-anaerober Mischinfektion [23].
- Bei Menschenbissen ist das Erregerspektrum größer – oft nachgewiesen werden hämolysierende Streptokokken, *Pasteurella*-Spezies, *Eikenella corrodens, Staphylococcus aureus* (einschließlich Methicillin-resistenter Staphylococcus aureus, MRSA) sowie verschiedene Anaerobier [25].

##### Merke

Das Erregerspektrum ist polymikrobiell und bei Menschenbissen breiter als bei Hunde- oder Katzenbissen.

#### Besondere infektiologische Aspekte

*Bartonella henselae* als Erreger der Katzenkratzkrankheit wird, wie der Name suggeriert, tatsächlich hauptsächlich bei Kratzverletzungen übertragen, selten bei Bissverletzungen [13].

Seltene, aber hochpathogene Erreger bei Hunde- und Katzenbissen sind ***Capnocytophaga*-Spezies**

Capnocytophaga-Spezies,

die ohne frühzeitige Therapie infolge diverser Pathogenitätsfaktoren kaum vom Immunsystem erkannt werden und oft lebensbedrohliche Infektionen auslösen. Klinisch unterscheiden sich die möglichen Wundinfektionen nicht von den durch andere Bakterien verursachten Lokalbefunden, es treten aber v. a. bei Patienten nach einer Splenektomie oder mit Alkoholabusus häufiger **septische Verläufe**

septische Verläufe

auf [26, 27]. Die kulturelle mikrobiologische Diagnostik aus infizierten Bisswunden ist essenziell [16], sowie auch die antibiotische Therapie, die jedoch beide grundsätzlich bei allen Bissverletzungen empfohlen werden [24, 28].

An eine mögliche Übertragung der virusbedingten Rabies (Tollwut) muss je nach Tier und Region unbedingt gedacht werden (in Deutschland insbesondere bei Bissen durch Fledermäuse, [29]). Die Erfragung des **Impfstatus**

Impfstatus

und ggf. die lebensrettende **Postexpositionsprophylaxe**

Postexpositionsprophylaxe

sind die entscheidenden Maßnahmen [16, 29]. Im Zweifelsfall muss je nach Grad der Exposition eine alleinige aktive bzw. eine zeitgleich aktive und passive Immunisierung durchgeführt werden ([2, 30]; Tab. 2). Die Verletzung eines Menschen durch ein tollwutkrankes, -verdächtiges oder -ansteckungsverdächtiges Tier sowie die Berührung eines solchen Tieres oder Tierkörpers sind nach § 6 des Infektionsschutzgesetzes meldepflichtig [31].

**Tab. 2:** Empfehlung zur Tollwutimmunisierung nach Art des Kontaktes. (Aus Rether [30])

Expositionsgrad	Art des Kontaktes	Immunprophylaxe
I	Berühren/Füttern Belecken intakter Haut	Keine
II	Oberflächliche Kratzer Belecken nichtintakter Haut	Aktive Immunisierung
III	Biss‑/Kratzwunden Schleimhautkontakt mit Speichel Belecken von Hautwunden Fledermauskontakt	Aktive und passive Immunisierung

Bei Menschenbissen/Faustschlagverletzungen muss zudem an die Möglichkeit der Übertragung von Hepatitis B, C und des Human Immunodeficiency Virus (HIV) gedacht und ggf. eine Postexpositionsprophylaxe durchgeführt werden [2]. Eine Abfrage des **Tetanus-Immunstatus**

Tetanus-Immunstatus

und ggf. Durchführung einer Auffrischungsimpfung sind obligat [32].

##### Merke

Es muss bei Tierbissen immer an die mögliche Übertragung von Tollwut und bei Menschenbissen an die von Infektionserkrankungen gedacht werden.

#### Erregernachweis und -identifikation

Im Rahmen des operativen Débridements sollten eine, besser mehrere, Gewebeproben (wenn nicht möglich: tiefer Abstrich) gewonnen werden. Gewebeproben sind Abstrichen in Sensitivität und Aussagekraft überlegen [9]. Die Transportzeit zum Labor muss möglichst kurz sein (idealerweise nicht länger als 2–4 h), weil Anaerobier bei längerer Sauerstoffexposition absterben [33]. Bei Anzeichen einer systemischen Infektion sollten **Blutkulturen**

Blutkulturen

(mindestens 2 Paare) gewonnen werden. Damit die Untersuchung sicher auf aerob *und* anaerob wachsende Erreger mit ausreichend langer Bebrütungsdauer (5 Tage) und eine adäquate mikrobiologische Bewertung des Befundes gewährleistet sind, muss dem Labor unbedingt der Hinweis auf die verursachende Bissverletzung gegeben werden.

##### Merke

Der mikrobiologische Nachweis sollte eher durch Gewebeproben als durch Abstriche erfolgen.

## Therapie

Eine evidenzbasierte Leitlinie für die Therapie existiert nicht. Bei äußerlich kleinen Verletzungen werden diese in ihrer Ausdehnung häufig unterschätzt. Große Verletzungen bedürfen einer interdisziplinären Therapie, möglicherweise zur Rekonstruktion mithilfe eines freien mikrochirurgischen Gewebetransfers (plastische Chirurgie).

### Antibiotikatherapie

#### Indikation

Die adäquate chirurgische Wundversorgung ist die wichtigste therapeutische Maßnahme [3]. Jede Bisswunde ist bakteriell kontaminiert, die Etablierung der Infektion dauert jedoch 8–12 h. Erst nach diesem Zeitraum sind typische **lokale Entzündungszeichen**

lokale Entzündungszeichen

sowie eine evtl. **regionale Lymphadenopathie**

regionale Lymphadenopathie

bewertbar [34]. Trotz der Therapie ist mit Infektionsraten beim Hundebiss von 10–30 %, beim Menschenbiss von 20–40 % und beim Katzenbiss von 30–60 % zu rechnen. Aufgrund der hohen Infektionsgefahr ist die Antibiotikatherapie in den meisten Fällen zusätzlich erforderlich [1, 9, 24, 28, 34, 35] und grundsätzlich für die Behandlung jeder Bisswunde zu empfehlen [28]. Nur in begründeten Ausnahmefällen wie z. B. einer erheblich verspäteten Vorstellung (> 2 bis 3 Tage) ohne jegliches Infektionszeichen kann auf eine Antibiotikatherapie verzichtet werden [34].

##### Merke

Auch wenn in der Literatur zum Stellenwert der präemptiven (einer sich bereits abzeichnenden Infektion zuvorkommenden) Antibiotikatherapie (somit keine originäre Prophylaxe) bei kurz zurückliegender Verletzung keine gute Evidenz besteht, ist die Antibiotikagabe grundsätzlich zu empfehlen.

#### Empfohlene Substanzen und Dosierung

Die kalkulierte oder empirische Antibiotikatherapie muss die häufigsten und relevantesten Erreger berücksichtigen, also in jedem Fall *Pasteurella*-Spezies, Staphylokokken, Streptokokken und Anaerobier. Die ideale Substanzkombination ist Amoxicillin + Clavulansäure (oral 3‑mal täglich 875 mg + 125 mg; i.v. 3‑mal 2,2 g) bzw. Ampicillin + Sulbactam (i.v. 3‑mal 3 g, keine orale Gabe, [36]).

Bei echter Penicillinallergie ist eine sinnvolle orale Therapie nicht mit einer Einzelsubstanz möglich. Das häufig im Fall einer Hautinfektionen angewandte Clindamycin ist bei Bissverletzungen nicht ausreichend, weil es keine Wirksamkeit gegen *Pasteurella-*Spezies hat, Chinolone hingegen haben keine Wirksamkeit gegen Anaerobier. Empfohlen wird eine Kombination aus Ciprofloxacin 2‑mal 500 mg oral (Hochdosis 2‑mal 750 mg; i.v. 2‑mal 400 mg, Hochdosis 3‑mal 400 mg) + Clindamycin 2‑mal 300 mg oral (Hochdosis 4‑mal 300 mg; i.v. 3‑mal 600 mg, Hochdosis 3‑mal 900 mg). Bei Menschenbissen mit manifester Infektion wird **Ertapenem**

Ertapenem,

einmal 1 g i.v., aufgrund der Wahrscheinlichkeit des Vorliegens von **Eikenella corrodens**

*Eikenella corrodens*

empfohlen [37].

##### Merke

Zur Therapie wird Amoxicillin + Clavulansäure p.o. empfohlen, Ampicillin + Sulbactam i.v. und bei Penicillinunverträglichkeit Ciprofloxacin + Clindamycin oral/i.v.

#### Applikationsdauer

Für die präemptive Antibiotikatherapie ist eine Applikationsdauer von 3 bis 5 Tagen ausreichend. Bei einer manifesten Infektion wird eine Dauer von 5 bis 10 Tagen empfohlen. In besonders ausgeprägten Fällen bzw. bei betroffenem bradytrophen Gewebe ist eine längere Therapie erforderlich (Sehnengewebe 2 bis 4 Wochen, Knochen 4 bis 6 Wochen oder ggf. länger, [9]).

### Besondere Komplikationen

#### Phlegmone

Hierbei handelt es sich um eine **bakterielle Infektion**

bakterielle Infektion,

die sich im Gegensatz zum abgegrenzten Abszess eher diffus entlang der anatomischen Strukturen im Bindegewebe ausbreitet und eine flächige Infektion hervorruft [14, 38]. Im Bereich der Hand können Finger, Hohl- oder Rückhand in unterschiedlichen Schweregraden betroffen sein [14].

#### Nekrotisierende Fasziitis

Auch für diese schwerwiegende Komplikation können Bissverletzungen auslösend sein. Häufigste Erreger bei nekrotisierender Fasziitis sind **A‑Streptokokken**

A‑Streptokokken

[39].

### Prinzipien der chirurgischen Sanierung

Grundlagen der chirurgischen Therapie sind das Ausschneiden der Bisskanäle, das Débridement und die ausgiebige Spülung zur Reduktion der Erregerlast [3]. Das Débridement soll die Entfernung des gesamten geschädigten, nekrotischen und infizierten Gewebes beinhalten. Sofern es möglich ist, sollten die anatomischen Grenzen (z. B. Faszien) respektiert werden. Das Débridement an den proximalen Extremitäten kann primär, wegen der vielen Möglichkeiten der späteren Weichteildefektdeckung, radikaler erfolgen als beispielweise an Gesicht und Händen [40, 41].

Zur Spülung können **Knopfkanülen**

Knopfkanülen

oder **Katheter**

Katheter

verwendet werden, mit deren Hilfe die Wunde entlang des Bisskanals bis in die Tiefe gespült wird (*Cave*: Keimverschleppung). Die Hochdruckspülung („jet lavage“) ist nicht zu empfehlen, da sie eine Verbreitung der Infektionserreger in die Gewebetiefe verursachen wird [42, 43, 44]. Als Spüllösung wird **physiologische Kochsalzlösung**

physiologische Kochsalzlösung

oder **Ringer-Lösung**

Ringer-Lösung

empfohlen. Diese Spüllösungen reduzieren mechanisch die Erregermenge; **superoxidiertes Wasser**

superoxidiertes Wasser

(SOD, Natriumhypochlorit) und **hypochlorige Säure**

hypochlorige Säure

(NaOCl + HOCl) wirken zudem bakterizid, ohne eine Zellschädigung zu verursachen [45, 46]. Besteht der Verdacht auf einen **Viruseintrag**

Viruseintrag,

sollte die Wunde mit einem viruziden Antiseptikum behandelt werden. Das Mittel der Wahl ist unverdünntes **Povidonjod**

Povidonjod

in alkoholischer Lösung, das gegenüber der wässrigen Lösung eine höhere Wirksamkeit und größere Tiefenwirkung zeigt. Jedoch stellt diese Therapie einen, eine besondere Aufklärung erfordernden, „off-label use“ dar [46]. Betroffene Gelenke werden gespült (als antiseptische Spüllösung schädigt nur SOD nicht den Knorpel) und mithilfe der **Drainagetherapie**

Drainagetherapie

weiterbehandelt bzw. wiederholt chirurgisch débridiert (Synovektomie). Ein **„second look“**

„second look“

ist nach 36–48 h indiziert [47].

#### Merke

Ausschneiden des Bisskanals, Débridement und Wundspülung sind die relevanten operativen Therapiemaßnahmen im Rahmen der Erstversorgung.

Bezüglich des **Wundverschlusses**

Wundverschlusses

von Bissverletzungen im Gesicht wird der primäre grobe, also leicht distanzierte Wundverschluss nach einem Débridement und Spülung empfohlen. **Narbenkorrekturen**

Narbenkorrekturen

sind risikoarm nach der Abheilung jederzeit durchführbar. Auch **Replantationen**

Replantationen,

z. B. nach traumatischer Amputation von Daumen und Langfinger [48], sowie **Rekonstruktionen**

Rekonstruktionen

werden primär durchgeführt [49]. Allgemeingültig scheint zu sein, dass ein primärer Wundverschluss 6–8 h, maximal 12 h nach der Verletzung noch möglich ist. Studien zu Hundebissverletzungen konnten zeigen, dass bei den primär genähten Wunden im Gesicht, selbst im Fall von mehrere Tage alten Wunden, im Vergleich zu Wunden mit einer Sekundärheilung keine erhöhte Rate der Wundinfektionen auftrat [50, 51]. Als Kontraindikationen gegen dieses „großzügige“ Vorgehen sind jedoch Katzenbissverletzungen an der Hand, Menschbissverletzungen sowie Wunden mit unklarer Tiefenausdehnung nach der Verletzung anzusehen [2, 47]. Dennoch ist trotz offener Wundbehandlung eine **grobe Wundadaptation**

grobe Wundadaptation

sinnvoll [2]. Größere Wunden können bei sicherer klinischer Beurteilbarkeit bis zur endgültigen plastischen Deckung oder dem Sekundärverschluss mithilfe der „negative pressure wound therapy“ (NPWT) temporär behandelt werden [52].

Nach Bissen durch **exotische Tiere**

exotische Tiere

(Schlangen, Spinnen) sollten die umgehende Vorstellung im Krankenhaus, die Ruhigstellung der betroffenen Extremität und der Kontakt zur **Giftnotzentrale**

Giftnotzentrale

zur Einschätzung der Toxizität erfolgen [2, 53].

#### Merke

Verzögert (> 12 h) vorgestellte Wunden sollten nicht primär verschlossen werden.

#### Fallbeispiel

**Anamnese**

Im November 2023 stellte sich ein 64-jähriger Patient in einem ihm nahegelegenen Krankenhaus vor. Sechs Tage zuvor wäre der Patient von einer anderen Person im Rahmen einer Auseinandersetzung in den linken Daumen gebissen worden. Die Bisswunden seien so tief gewesen, dass sie initial stark geblutet hätten. Erst verspätet erfolgte im Verlauf die Vorstellung in einer Notaufnahme mit progredienten Schmerzen und Bewegungseinschränkungen. Infolge fehlender handchirurgischer Expertise war die Verlegung initiiert worden.

**Klinischer Befund**

**Abb. 7:**
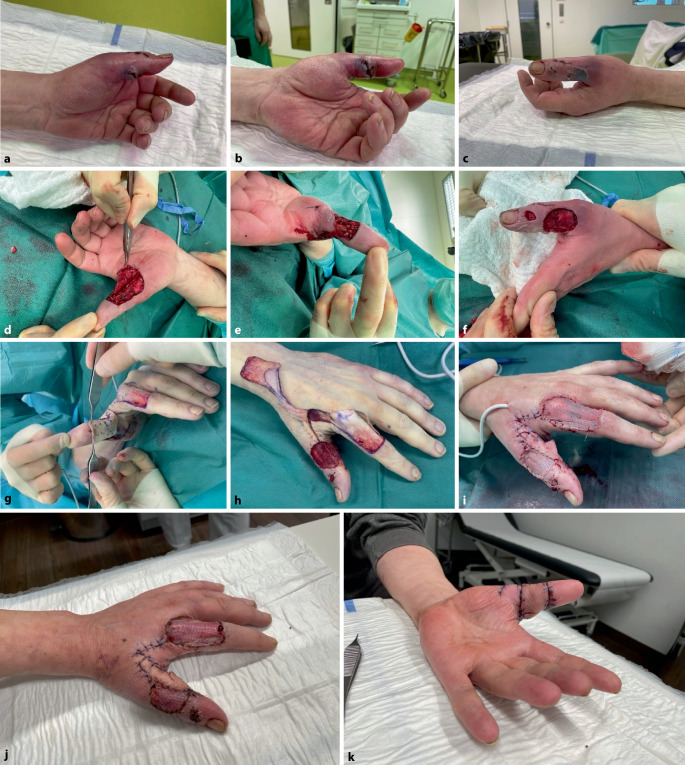
**a–c** Menschenbissverletzung: massive Phlegmone des gesamten linken Daumens mit Bisswunden palmar- und ulnarseitig, jeweils proximal des Interphalangealgelenkes. **d–f** Darstellung der sauberen Wundverhältnisse nach 2‑maligem Wund-Débridement und Spülung. **g,** **h** Intraoperativer Befund nach einer Lappenhebung: Skizzieren einer Hautinsel an der Grundgliedstreckseite des Digitus manus II, S‑förmiger Hautschnitt im ersten Interdigitalraum radial, Identifikation der Arterie und Präparieren eines Faszien-Gefäß-Nerven-Stiels unter Einschluss der Faszie des M. interosseus, Umschneiden des Insellappens am Digitus manus II, Heben und Mobilisation der Lappenplastik, Schwenken des Lappens unter die vorher untertunnelte Haut und Einnähen es Lappens, Aufbringen der Spalthaut bei Entnahme des linken Oberschenkels. **i** Postoperativer Befund nach der Foucher-Lappenplastik. **j,** **k** Entlassungsbefund mit gut eingewachsener Lappen- und Spalthautplastik am achten postoperativen Tag

Es zeigte sich eine ausgeprägte Phlegmone des gesamten linken Daumens (Abb. 7a–c). Die Bisswunden lagen palmar und ulnar, jeweils proximal des Interphalangealgelenks. Im Bereich der Wunden war die Haut bereits nekrotisch mit Epitheliolysen. Sonographisch bestand ein hochgradiger Verdacht auf die Entwicklung einer Phlegmone. Radiologisch konnte eine knöcherne Beteiligung ausgeschlossen werden.

**Therapie**

Bei der notfallmäßigen operativen Intervention wurden die Daumenphlegmone beugeseitig eröffnet, exploriert und radikal débridiert sowie die Beugesehnenscheide gespalten und gespült. Zur temporären Weichteildeckung wurde ein alloplastischer multifunktionaler polymerer Membranverband eingesetzt. Zwei Tage später erfolgte ein programmierter *second look*, mit erneutem Wund-Débridement, Spülung und Wechsel der polymeren Wundauflage mit Schwamm (Abb. 7d–f). In beiden Operationen wurden Gewebeproben zur mikrobiologischen Diagnostik entnommen; diese ergaben den mikrobiologischen Nachweis von *Streptococcus pyogenes* und *Streptococcus gordonii*. Die bereits perioperativ begonnene antimikrobielle Therapie mit Ampicillin + Sulbactam konnte dem Antibiogramm entsprechend resistenzgerecht fortgeführt werden und wurde am 8. postoperativen Tag bei unauffälligen Weichteilbefunden beendet. Vier Tage nach dem *Second look* konnte aufgrund der sauberen und reizfreien Wundverhältnisse die geplante Weichteilrekonstruktion mithilfe einer neurovaskulär gestielten Foucher-Lappenplastik erfolgen (Abb. 7g–i).

**Verlauf und Nachbehandlung**

Der auf die Mesh gelegte polymere Membranverband mit Schwamm wurde nach 5 Tagen entfernt. Nach dem Drainagezug erfolgte die Entlassung in die Häuslichkeit 8 Tage nach der letzten Operation mit vitalem Lappen und gutem Anwachsen der Spalthaut (Abb. 7j, k; Fadenzug nach 14 Tagen).

## Fazit für die Praxis

- Hundebissverletzungen stellen die häufigste Form der Bissverletzung dar, verlaufen aber weniger komplikativ als Katzen- oder Menschenbisse.
- Die Infektionsraten nach einem Hundebiss betragen 10–30 %, nach einem Menschenbiss 20–40 % und nach einem Katzenbiss 30–60 %.
- Eine ausführliche Anamnese mit Erfragung des Impfstatus von Beißverursacher und Bissopfer ist essenziell.
- Zur Wundbeschreibung gehören Ausmaß des Schadens, Beteiligung von Strukturen, Vorhandensein von Fremdkörpern und Beweglichkeit betroffener Gelenke.
- Insbesondere Katzenbisse können sich klinisch wenig imponierend präsentieren, haben aber ein hohes Infektions- und Komplikationsrisiko.
- Ein mikrobiologischer Nachweis durch Gewebeproben sollte für eine suffiziente Antibiotikatherapie immer angestrebt werden.
- Die kalkulierte Antibiotikatherapie sollte mit Amoxicillin + Clavulansäure oder Ampicillin + Sulbactam erfolgen.
- Die Abtragung der Nekrosen in allen Gewebsschichten und die ausgiebige Spülung zur Reduktion der Erregerlast stellen die wesentlichen Maßnahmen im Rahmen der chirurgischen Therapie dar.
- Verletzte tiefer liegende Strukturen, wie Sehnen, Knorpel und Gelenke, sollten identifiziert und ggf. chirurgisch saniert werden.

## CME-Fragebogen

Die Erfragung des Impfstatus nach Bissverletzungen spielt eine wichtige Rolle. Worauf sollte dabei geachtet werden?

Bei sofortiger antibiotischer Therapie ist der Impfstatus sekundär.

Eine Tetanusauffrischimpfung ist nicht simultan mit einer Tollwutimpfung durchführbar.

Patienten nach einer Splenektomie sollten unmittelbar gegen Tetanus geimpft werden.

Im Zweifelsfall muss eine aktive oder zusätzlich passive Immunisierung gegen Tetanus erfolgen.

Eine Überprüfung des Zeckenimpfschutzes ist nach Hundebissen obligat.

Wodurch zeichnen sich Hundebissverletzungen aus?

Sie führen in der Regel zu punktförmigen Verletzungen.

Sie führen zu Riss‑/Quetschwunden.

Sie führen bevorzugt zu Verletzungen an der unteren Extremität.

Sie führen zu schwerwiegenden Verletzungsmustern mit hoher Letalität.

Sie führen häufig zur Tollwuterkrankung.

Ein 3‑jähriges Mädchen wird durch ihre Eltern in der Rettungsstelle mit einer Katzenbissverletzung an der rechten Hand vorgestellt. Was kennzeichnet Katzenbissverletzungen?

Sie führen zu halbmondförmigen Verletzungen der Haut.

Die Füße sind gehäuft betroffen.

Die Infektionsrate der Wunden ist geringer als bei Hunden.

Sie führen infolge einer Lymphangiosis zu Rötung, Ödem und Schmerz.

Typischerweise kommt es dabei zu Quetschverletzungen.

Was versteht man unter der „Fight-bite-clenched-fist-Verletzung“?

Die direkte Bissverletzung des Weichteilgewebes durch Quetschung zwischen den Zähnen

Die typische Bissverletzung des Kindes

Die Bissverletzungen im Bereich der Brust und der Arme

Die indirekte Bissverletzung durch den Schlag der Faust gegen die Zähne

Die Bissverletzungen durch Schlangen und Nagetiere

Wie sollte die mikrobiologische Diagnostik nach einer Bissverletzung durchgeführt werden?

Ein oberflächlicher Abstrich sollte unverzüglich an das Labor gesendet werden.

Gewebeproben sind Abstrichen überlegen.

Eine ausreichend lange Bebrütungsdauer von 3 Tagen sollte eingehalten werden.

Bei Schmerzen an der Bissstelle ist die Gewinnung einer Blutkultur indiziert.

Die Untersuchung auf aerobe Erreger ist ausreichend.

Welche Antibiotikatherapie ist zur Behandlung bei Bissverletzungen empfohlen?

Bei Penicillinallergie ist Clindamycin als Einzelsubstanz das Mittel der Wahl.

Es kann z. B. Amoxicillin + Clavulansäure oral gegeben werden.

Chinolone haben eine gute Wirksamkeit gegen Anaerobier.

Die orale Gabe von Ampicillin + Sulbactam ist der i.v.-Therapie überlegen.

Nach Pferdebissen erfolgt die orale Gabe von Ertapenem.

Was stellt die Grundlage der chirurgischen Therapie nach Bissverletzungen dar?

Im Falle einer persistierenden Infektion über 3 Tage sollte eine operative Revision erfolgen.

Wichtig sind ein ausgiebiges Wund-Débridement und eine ausgiebige Spülung.

Die Hochdruckspülung („jet lavage“) sollte bevorzugt werden.

Gelenke sollen mit unverdünnter Jodlösung gespült werden.

Ein primärer Wundverschluss ist bis zu 48 h nach einer Bissverletzung durchführbar.

Auf welche Art und Weise sollten das Wund-Débridement und die weitere Versorgung nach Bissverletzungen erfolgen?

Das Débridement sollte unabhängig von der Lokalisation gleich radikal durchgeführt werden.

Gelenke und Sehnen sollten vom Débridement ausgespart werden.

Veraltete Wunden (> 12 h) sollten in der Regel nicht primär verschlossen werden.

Bei Bissverletzungen im Gesicht wird eine offene Wundbehandlung angestrebt.

Die Replantation eines Fingers nach traumatischer Amputation erfolgt sekundär.

Worauf sollte bei Bissverletzungen durch exotische Tiere geachtet werden?

Bissverletzungen durch Schlangen sollten ohne Exzision direkt vernäht werden.

Spinnenbisse erfordern häufig eine Amputation.

Eine Wundspülung ist kontraindiziert, um das Gift nicht in die Tiefe zu verschleppen.

Im Fall eines Bisses sollte unbedingt die Notrufzentrale kontaktiert werden.

Die verletzte Extremität sollte sofort weiterbewegt werden, um Versteifungen zu vermeiden.

Ein männlicher 64-jähriger Patient stellt sich mit einer massiven Menschenbisswunde und Umgebungsrötung im Bereich des Daumens der linken Hand vor. Neben der Antibiotikatherapie ist eine Weichteilsanierung mit Rekonstruktion eines Weichteildefektes notwendig. Eine Foucher-Lappenplastik wird geplant. Was versteht man darunter?

Einen gestielten neurovaskulären Insellappen der 1. dorsalen Metakarpalarterie (DMCA), der zur Defektdeckung am Daumen verwendet wird

Einen speziellen Schwenklappen, bei dem das Gewebe so mobilisiert wird, dass sowohl der primäre Defekt als auch der Entnahmedefekt gedeckt ist

Einen Transpositionslappen, der zur Defektdeckung dient und zufallsversorgt ist

Einen Schwenklappen zur Deckung von Hautdefekten, der sich als Nahlappen, Hautlappen als auch als zufallsversorgter Lappen einordnen lässt

Einen Nahlappen, bei dem ein gestielter Lappen aus der Entnahmestelle linear in den Defekt verschoben wird
